# A latent profile analysis of attitudes towards artificial sweetener consumption among adults in Saudi Arabia

**DOI:** 10.3389/fpubh.2026.1841996

**Published:** 2026-10-09

**Authors:** Abeer A. Aljehani, Buthaina M. Aljehany, Eman A. Abduljawad, Sarah A. Alasmari, Afnan H. Saaty, Dalal A. Alkhudhayri, Nada Benajiba, Howeida Abusalih

**Affiliations:** 1 Food and Nutrition Department, Human Sciences and Design Faculty, King Abdulaziz University, Jeddah, Saudi Arabia; 2 Public Health Department, College of Health Sciences, Saudi Electronic University, Riyadh, Saudi Arabia; 3 Department of Home Economic, Prince Sattam Bin Abdulaziz University, Alkharj, Saudi Arabia; 4 Joint Research Unit in Nutrition and Food, RDC-Nutrition AFRA/IAEA, Ibn Tofail University-CNESTEN, Kenitra, Rabat, Morocco; 5 Department of Health Sciences, College of Health and Rehabilitation Sciences, Princess Nourah bint Abdulrahman University, Riyadh, Saudi Arabia

**Keywords:** adults, artificial sweeteners, attitudes, latent profile analysis, Saudi Arabia

## Abstract

**Background:**

Artificial sweeteners are increasingly used as substitutes for added sugars, particularly in regions undergoing rapid dietary changes such as Saudi Arabia. However, little is known about how attitudes toward these products vary across individuals and how these attitudes relate to consumption patterns. This study aimed to identify distinct attitudinal profiles toward artificial sweeteners among Saudi adults and to examine their association with consumption behaviors.

**Methods:**

A cross-sectional study was conducted between November 2023 and February 2024 among 386 adults in Saudi Arabia. Data was collected using an online questionnaire assessing socio-demographic characteristics, attitudes toward artificial sweeteners using the Artificial Sweetener Attitude Scale (AASS), and consumption frequency. Latent Profile Analysis (LPA) was applied to identify subgroups based on attitude dimensions.

**Results:**

Three profiles emerged—benefit-oriented, risk-sensitive, and neutral—differing in perceived benefits, risks, trust in regulatory authorities, and preference for natural foods. Consumption followed a clear gradient, highest among benefit-oriented and lowest among risk-sensitive participants (*p* = 0.004). Educational level was the only socio-demographic variable associated with profile membership at the bivariate level (*p* = 0.035), but this did not persist after adjustment for age, sex, income, region, marital status, and employment status; age and region instead emerged as independent predictors. Lower consumption among risk-sensitive participants remained significant after full adjustment (B = −6.51, *p* = 0.002), not attributable to demographic confounding.

**Conclusion:**

Distinct attitudinal profiles are associated with artificial sweetener consumption among Saudi adults, robust to demographic adjustment, whereas education alone does not independently explain profile membership. The observed alignment between perceptions and behavior underscores the importance of moving beyond one-size-fits-all strategies. These findings support tailored public health interventions and risk communication over one-size-fits-all strategies in Saudi Arabia.

## 1 Introduction

The global burden of non-communicable diseases (NCDs) has reached unprecedented levels, posing a defining challenge to public health systems worldwide. NCDs are currently responsible for approximately 43 million deaths annually, accounting for nearly 75% of all global deaths, with dietary risk factors—particularly excessive sugar intake—identified as major contributing drivers (1). The scale of associated metabolic conditions is equally alarming: approximately 2.5 billion adults worldwide (43%) are affected by overweight or obesity, including nearly 890 million living with obesity (2, 3), while diabetes alone affects around 589 million adults globally, representing 11% of the adult population (4). In response to this mounting evidence, international health authorities have recommended since decade ago substantial reductions in free sugar intake as a central strategy to curb the growing burden of diet-related diseases (5). These recommendations have reshaped the food and beverage landscape. Driven by both regulatory pressure and consumer demand for healthier options, the industry has increasingly replaced added sugars with artificial sweeteners—high-intensity, low-calorie substances such as aspartame, acesulfame potassium, and sucralose—that deliver sweetness with minimal caloric contribution (6). Approved by regulatory bodies including the U. S. Food and Drug Administration and the European Food Safety Authority, and assigned acceptable daily intake values to ensure safety within defined limits (7), these sweeteners have become deeply embedded in the modern food supply. In the United States alone, national dietary surveys have documented substantial and growing consumption of low-calorie sweeteners among both children and adults (8), reflecting a broader global trend toward their normalization as a dietary tool.

Yet the health credentials of artificial sweeteners are far from settled. Despite their widespread adoption for weight management and glycemic control, the evidence on their long-term health effects remains mixed and increasingly contested (9). While randomized controlled trials have suggested that replacing sugar with non-sugar sweeteners may modestly reduce short-term energy intake and body weight under controlled conditions, observational studies have frequently reported inconsistent or even adverse associations with obesity, cardiometabolic disease, and mortality (10, 11). This discrepancy has generated considerable scientific debate regarding the interpretation of existing evidence and the methodological limitations inherent in artificial sweetener research, including reverse causality, residual confounding, and variability across sweetener types and consumption patterns (11).

A systematic review and meta-analysis by Azad et al. (12) identified potential associations between non-nutritive sweetener consumption and increased body mass index and other cardiometabolic risk factors. More strikingly, analysis of the large French NutriNet-Santé prospective cohort revealed that total artificial sweetener intake was associated with an elevated risk of cardiovascular disease overall (hazard ratio = 1.09; 95% CI, 1.01–1.18), with an even stronger association observed for cerebrovascular disease (HR = 1.18; 95% CI, 1.06–1.31); differential effects were also observed for specific sweeteners including aspartame, acesulfame potassium, and sucralose (13, 14). At the mechanistic level, a landmark study published in *Nature* demonstrated that consumption of commonly used artificial sweetener formulations—including saccharin, sucralose, and aspartame—was associated with the development of glucose intolerance through compositional and functional alterations to the gut microbiota, with these effects observed in both animal models and healthy human subjects (12). More recently, emerging evidence has continued to raise questions regarding the potential effects of certain non-sugar sweeteners on appetite regulation, insulin response, microbiome composition, and long-term cardiometabolic health, although findings remain inconsistent across study designs and populations (15, 16). Importantly, not all evidence has been unfavorable. Several controlled intervention studies and systematic reviews have reported that replacing sugar-sweetened beverages with low- or no-calorie sweetened alternatives may contribute to modest reductions in body weight and energy intake, particularly when incorporated into broader calorie-reduction strategies (10). Consequently, the current scientific landscape is characterized less by consensus than by ongoing debate concerning the balance between potential benefits, behavioral compensation, and long-term health uncertainties.

Reflecting the weight of this accumulating evidence, the World Health Organization has issued guidance in 2023 advising against the routine use of non-sugar sweeteners for weight control, citing limited and inconsistent evidence of long-term benefit (16). Taken together, these findings challenge the prevailing assumption that artificial sweeteners represent a straightforward and risk-free alternative to sugar, and highlight the urgent need to better understand how consumers perceive and respond to this evolving scientific landscape.

This question is especially pertinent in the Gulf Cooperation Council (GCC) region, where a rapid nutrition transition—marked by the widespread proliferation of ultra-processed and sugar-reduced food products—has coincided with some of the highest rates of obesity and metabolic disease globally (17–19). In this context, artificial sweeteners have emerged as a common dietary strategy among health-conscious consumers seeking to reduce sugar intake and manage body weight. In the United Arab Emirates, a study among female university students found that 24% consumed artificially sweetened foods and beverages, while 15.1% used sweeteners in tablet or powder form, with weight control and perceived health benefits as the primary motivations (20). In Saudi Arabia, regional data from the Tabuk area indicate that artificial sweetener consumption is significantly associated with sociodemographic characteristics, including age, gender, and educational level (21). More recently, a cross-sectional study of 386 Saudi adults reported that approximately 42% were regular users of artificial sweeteners, with 65% citing weight management as their primary motivation and nearly 74% perceiving these products as supportive of a healthy lifestyle; female gender and higher educational attainment emerged as significant predictors of use (22). These figures reveal a population that is actively and increasingly incorporating artificial sweeteners into everyday dietary practice—yet one whose attitudes toward the risks and benefits of these substances have not been systematically examined.

Consumer behavior around artificial sweeteners is not shaped by knowledge or demographics alone—it reflects a mix of attitudes that traditional research methods struggle to fully capture. For example, the same person might see artificial sweeteners as helpful for weight control, yet still worry about their long-term safety, while only partially trusting the regulatory bodies that approve them. These attitudes do not exist in isolation; they interact with each other in ways that vary from person to person. Conventional statistical approaches, such as regression analysis or ANOVA, examine how individual variables relate to each other across a population, but they assume a degree of similarity among people that rarely holds true in practice. As a result, they tend to miss the combinations of attitudes that actually drive consumer behavior (23). They can identify what predicts an attitude on average, but they cannot reveal the distinct configurations of beliefs, risk perceptions, and trust that co-exist within different consumer subgroups. This is a critical analytical gap, because it is precisely this heterogeneity in attitudinal profiles—not mean-level differences—that is likely to drive variation in consumption behavior and receptiveness to health communication. Hence, person-centered analytical approaches offer a principled solution to this problem. Latent Profile Analysis enables the identification of distinct subgroups of individuals who share similar patterns across multiple attitude dimensions simultaneously, without imposing *a priori* assumptions about the structure of responses in the population (24, 25). By mapping how perceptions of risk, perceived benefit, and regulatory trust co-occur within individuals, LPA moves beyond aggregate comparisons to reveal the latent attitudinal structure that characterizes real-world consumer populations—an approach that has demonstrated particular utility in health and behavioral research where population heterogeneity is the rule rather than the exception (25). Despite the clear relevance of this approach, no study to date has applied Latent Profile Analysis to characterize attitudinal profiles toward artificial sweeteners in the context of Saudi Arabia, nor examined how such profiles relate to actual consumption behaviors. This study aims to fill this gap in two ways. First, it seeks to identify distinct groups of consumers in Saudi Arabia based on how they think and feel about artificial sweeteners—specifically their perceptions of risk and benefit, and how much they trust regulatory authorities. Second, it examines whether belonging to one of these groups is linked to differences in how much artificial sweeteners people actually consume. By focusing on real attitudinal patterns rather than population averages, the findings are intended to provide practical, evidence-based guidance for public health messaging and food policy in Saudi Arabia and the wider GCC region.

## 2 Methodology

### 2.1 Study design and participants

A cross-sectional study was conducted between November 2023 and February 2024 among adults aged 18 years and older residing in the Kingdom of Saudi Arabia. Eligibility criteria included Saudi residents who were able to read and understand Arabic and who agreed to participate voluntarily in the study. Individuals diagnosed with eating disorders or medical conditions that may influence artificial sweetener intake, such as phenylketonuria, were excluded.

The study protocol received ethical approval from the Human Research Ethics Committee at King Abdulaziz University (Reference No. 453–23). All participants were informed about the objectives of the study, the voluntary nature of participation, and their right to withdraw at any stage without consequences. An electronic informed consent form was provided prior to participation. No personal identifiers were collected, ensuring the anonymity and confidentiality of all responses. Participation posed no known risks, and no financial incentives were provided. To enhance data integrity, the online questionnaire was configured to allow only one response per participant, reducing the likelihood of duplicate submissions. Additionally, responses were screened for completeness before inclusion in the final dataset.

### 2.2 Sample size and sampling strategy

The minimum required sample size was determined *a priori* using Cochran’s formula for cross-sectional prevalence studies involving a large population (26): *n* = Z^2^ x p(1-p)/e^2^, where *n* is the required sample size, *Z* is the z-score corresponding to the desired confidence level (1.96 for a 95% confidence interval), *p* is the estimated prevalence of the characteristic of interest, and *e* is the acceptable margin of error (0.05). In the absence of nationally representative data on the prevalence of artificial sweetener consumption among Saudi adults, a conservative estimate of *p* = 0.50 was adopted, as this value maximizes the required sample size and therefore yields the most statistically conservative estimate regardless of the true population proportion (27). This is a widely accepted convention in cross-sectional health behavior research conducted in settings where population-specific prevalence data are unavailable (28, 29). Applying these parameters yields a minimum required sample size of 385 participants. Recruitment was conducted using a convenience sampling approach.

To ensure data integrity and the reliability of respondents’ answers, several quality control procedures were implemented. The online questionnaire was configured to allow only one response per participant (via IP and account restrictions), reducing the likelihood of duplicate submissions. Additionally, all responses were screened for completeness and straight lining (providing identical answers across all Likert items) prior to inclusion. While the use of self-reported data introduces potential recall or social desirability bias, these measures were taken to maximize the accuracy of the final dataset.

### 2.3 Data collection procedure

Data was collected through a structured online questionnaire developed using Google Forms. The survey link was disseminated through multiple digital platforms, including social media channels such as WhatsApp and Twitter, as well as institutional email networks associated with King Abdulaziz University.

The questionnaire was administered in Arabic to ensure accessibility and cultural relevance for Saudi participants. The original items were initially prepared in English and subsequently translated into Arabic. To ensure linguistic accuracy and conceptual equivalence, the Arabic version was independently back-translated into English by bilingual experts in nutrition and public health.

### 2.4 Study instrument

The questionnaire consisted of three main sections designed to capture socio-demographic information, attitudes toward artificial sweeteners, and patterns of artificial sweetener consumption.

*Socio-demographic and health characteristics*: The first section collected demographic and health-related information through 13 questions. Variables included age, sex, place of residence, nationality, educational level, monthly household income, marital status, and self-reported health status.

*Attitudes toward artificial sweeteners*: Participants’ perceptions and attitudes were assessed using the Artificial Sweetener Attitude Scale (AASS), a previously validated instrument comprising 23 items grouped into five dimensions: acceptance, perceived risk, perceived benefits, trust in regulatory authorities, and motivation for natural foods (23, 30). The Acceptance dimension (6 items) measures participants’ willingness to consume foods containing artificial sweeteners and the degree to which they consider sweetener content a relevant concern in their food choices. Items assess whether participants actively check product labels for artificial sweetener content, whether they are bothered by the presence of artificial sweeteners in their foods, whether they can accept foods containing artificial sweeteners, and whether they consider sweetener-related concerns worthy of attention (e.g., “I can accept that certain foods contain artificial sweeteners”; “It does not bother me if my foods contain artificial sweeteners”; “I think it is unimportant to check on the packaging whether a food contains artificial sweeteners”; “I have more important things to do than worry about artificial sweeteners”). The Perceived Risk dimension (5 items) assesses the extent to which participants perceive artificial sweeteners as a health hazard. Items cover general feelings of unease when thinking about artificial sweeteners, concern about their effects on the body, and beliefs that certain artificial sweeteners are unhealthy or harmful to human health (e.g., “When I think of artificial sweeteners, I get an uneasy feeling”; “I am worried about what effects artificial sweeteners could have on my body”; “I think that artificial sweeteners are a risk to human health”). The Perceived Benefits dimension (5 items) evaluates participants’ beliefs about the health and practical advantages of artificial sweetener use. Items address whether artificial sweeteners help reduce caloric intake, whether they bring personal benefits to the consumer, whether they enable the production of diet products, and whether they allow for enjoyment of sweet foods without negative consequences (e.g., “Artificial sweeteners allow for a reduction of unnecessary calories”; “The use of artificial sweeteners brings benefits for me personally”; “Artificial sweeteners allow for indulgence without regret”).

The Trust in Regulatory Authorities dimension (3 items) examines participants’ confidence in food safety regulatory bodies—operationalized in the Saudi context as the Saudi Food and Drug Authority (SFDA)—to protect consumer health and ensure the safe use of artificial sweeteners. Items assess general trust in regulatory authorities, trust in the steps taken to protect consumers’ health, and trust in relation to the specific licensing and control of artificial sweeteners in food products (e.g., “I think that you can trust the regulators”; “I trust the regulators to make sure every necessary step is taken to protect consumers’ health”; “I trust the regulators in relation to the licensing and control of artificial sweeteners in foods”).

The Motivation for Natural Foods dimension (6 items) assesses participants’ preference for natural food products over those containing artificial sweeteners or other additives. Items cover beliefs about the health superiority of natural foods, the perceived taste advantage of natural foods, willingness to pay a higher price for natural products, the perceived nutritional quality of natural foods, a general sense of wellbeing associated with eating natural foods, and active attention to the naturalness of products during grocery shopping (e.g., “Natural foods are better for my health”; “Natural foods taste better than other foods”; “I gladly pay a higher price for natural foods”; “I pay attention during grocery shopping to ensure that the foods are as natural as possible”). All items were rated on a six-point Likert scale ranging from 1 [very strongly disagree) to 6 (very strongly agree) (the complete bilingual (English and Arabic) instrument is provided as Supplementary material]. The total possible score ranged from 23 to 138, with higher scores reflecting more favorable attitudes toward artificial sweeteners. Scores were also calculated separately for each dimension to capture distinct aspects of participants’ perceptions. Scores were analyzed both at the overall and subscale levels to capture distinct attitudinal dimensions.

*Artificial sweetener consumption frequency*: The third section evaluated the frequency of artificial sweetener consumption and was adapted from previously published research (22, 30). Participants were asked to report how frequently they consumed products containing artificial sweeteners, including sugar-free soft drinks and beverages sweetened with artificial sweeteners such as tea or coffee. Participants were asked to report how frequently they consumed products containing artificial sweeteners over the previous month. The consumption frequency section comprised 18 food and beverage items organized across five categories: beverages (7 items), cooked food (2 items), spreadable foods (2 items), dairy products (3 items), and biscuits and sweets (4 items). For each item, participants selected from 10 predefined frequency categories: never, once a month, 2–3 times a month, 1–2 times a week, 3–4 times a week, 5–6 times a week, once a day, 2–3 times a day, 4–5 times a day, and 6 times or more a day. For analytical purposes, each frequency category was assigned an ordinal numerical value from 0 to 10, reflecting increasing consumption frequency, where 0 = never and 10 = six times or more per day. Individual item scores were subsequently summed to generate an overall consumption frequency score. Given that the scale comprised 18 items each scored from 0 to 10, the possible total score ranged from a minimum of 0 (never consuming any product) to a maximum of 180 (consuming all 18 products at the highest frequency category), with higher scores indicating greater overall artificial sweetener consumption. The classification into low, moderate, and high consumption levels was based on the theoretical range of the total possible score (minimum–maximum scoring range). Specifically, cut-off points were defined by dividing the total possible score range into three equal intervals, allowing for an *a priori* and standardized categorization of consumption intensity. This approach was adopted in the absence of established or validated thresholds for artificial sweetener consumption and was intended to provide a consistent and interpretable framework for descriptive groups. Hence, categorization was determined as follows: 0–60 for low consumption, 61–120 for moderate consumption, and 121–180 for high consumption (Figure 1).

**Figure 1:**
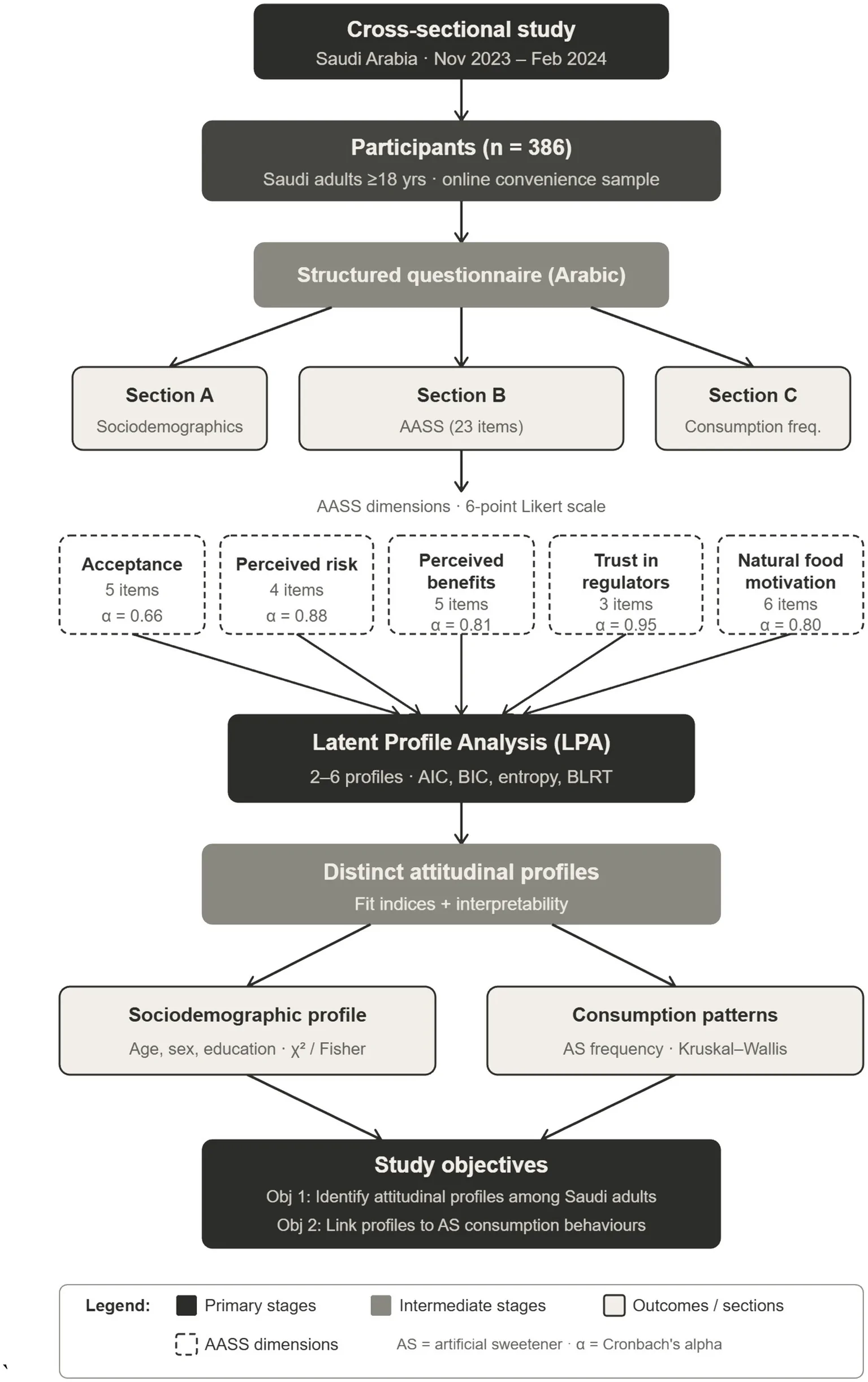
Conceptual framework and study flow diagram. AASS, Artificial Sweetener Attitude Scale; LPA, Latent Profile Analysis; AIC, Akaike Information Criterion; BIC, Bayesian Information Criterion; BLRT, Bootstrap Likelihood Ratio Test; AS, artificial sweetener. Cronbach’s alpha (α) values are reported per AASS dimension.

### 2.5 Reliability assessment

The internal consistency of the attitude scale was evaluated using Cronbach’s alpha coefficients. High reliability was observed for several dimensions, including perceived risk (α = 0.88), perceived benefits (α = 0.81), trust in regulators (α = 0.95), and motivation for natural foods (α = 0.80). The acceptance dimension demonstrated acceptable reliability with an alpha value of 0.66. It should be noted that the overall Cronbach’s alpha for the full 23-item AASS (α = 0.68) is not an appropriate primary reliability indicator for this instrument, given its multidimensional structure. For multidimensional scales, overall alpha is known to underestimate reliability when subscales measure conceptually distinct constructs with low inter-subscale correlations, as is the case here (31, 32). The analytically relevant reliability estimates are therefore those reported at the subscale level, which ranged from acceptable to excellent (α = 0.80–0.95) across four of the five dimensions. The sole reliability concern is the acceptance subscale (α = 0.66, 5 items), which falls marginally below the conventional threshold of 0.70; however, this value is within the range commonly observed for short attitudinal subscales in exploratory research (33, 34).

### 2.6 Statistical analysis

All statistical analyses were performed using R statistical software (version 4.4.0; R Foundation for Statistical Computing, Vienna, Austria). The normality of continuous variables was assessed using the Shapiro–Wilk test together with visual inspection of quantile–quantile (Q–Q) plots. As the normality assumption was not satisfied, continuous variables were summarized using median and interquartile range (IQR). Comparisons between groups for continuous variables were performed using the Kruskal–Wallis test, while categorical variables were summarized as frequencies and percentages. Differences between proportions were examined using the Chi-square test or the Fisher–Freeman–Halton exact test, when appropriate. Associations between continuous variables were explored using Spearman’s rank correlation coefficient. All statistical tests were two-sided, and *p*-values less than 0.05 were considered statistically significant.

#### 2.6.1 Latent profile analysis

To identify homogeneous subgroups of participants based on their attitudes toward artificial sweeteners, a Latent Profile Analysis (LPA) was conducted using the *tidyLPA* package in R (35). LPA is a person-centered statistical approach derived from finite mixture modeling that identifies unobserved subgroups of individuals sharing similar response patterns on continuous indicator variables—in this study, the five subscale scores of the Artificial Sweetener Attitude Scale (AASS): acceptance, perceived risk, perceived benefit, trust in regulatory authorities, and motivation for natural foods (25, 36).

Models specifying two to six latent profiles were estimated and compared across multiple covariance structures, as recommended by Masyn et al. (37). Specifically, four model specifications were evaluated: (1) equal variances and zero covariances, (2) varying variances and zero covariances, (3) equal variances and equal covariances, and (4) varying variances and varying covariances. The model allowing varying variances and zero covariances was selected as the final specification for the following reasons. First, allowing variances to vary freely across profiles is theoretically justified in attitudinal research, where different consumer subgroups are expected to differ not only in their mean levels of perceived risk, benefit, and trust, but also in the degree of within-profile heterogeneity on each dimension—that is, some profiles may be more internally homogeneous than others (25). Second, the assumption of zero covariances—meaning that the indicator variables are assumed to be uncorrelated within profiles after accounting for profile membership—was retained on both statistical and parsimony grounds: this specification produces a more parsimonious model than freely estimating covariances, reduces the risk of overfitting in moderate sample sizes such as the present study (*n* = 386), and is the most widely adopted specification in applied LPA research in health and behavioral sciences (38). Third, among the covariance structures evaluated, the varying variances and zero covariances model produced the best balance of model fit and parsimony, as reflected in lower BIC values and acceptable entropy relative to more complex specifications (39).

The optimal number of profiles within the selected covariance structure was determined based on a combination of statistical fit indices and substantive interpretability. Statistical criteria included the Akaike Information Criterion (AIC), Bayesian Information Criterion (BIC), sample-size-adjusted BIC (aBIC), entropy, minimum posterior classification probabilities, and the Bootstrap Likelihood Ratio Test (BLRT). Lower AIC and BIC values indicate better model fit relative to model complexity; entropy values closer to 1.00 indicate higher classification certainty; and minimum posterior classification probabilities of ≥ 0.70 indicate adequate classification accuracy (37). In addition to these statistical criteria, the theoretical interpretability and practical meaningfulness of each profile solution were considered in the final model selection decision, consistent with best practice recommendations for applied LPA (25). Following model selection, the latent profiles were compared with respect to sociodemographic characteristics and levels of artificial sweetener consumption. Group differences were assessed using the Chi-square test or Fisher–Freeman–Halton exact test for categorical variables, and the Kruskal–Wallis test for continuous variables, with a significance threshold of *p* < 0.05.

*Multivariable analysis of profile membership and consumption*: To evaluate whether the bivariate associations reported above were independent of other demographic characteristics, two multivariable models were fitted. First, a multinomial logistic regression was used to examine whether educational attainment independently predicted latent profile membership, with the Neutral profile (the modal class) specified as the reference outcome category and sex, age group, educational level, monthly income, region of residence, marital status, and employment status entered simultaneously as covariates. Second, a multiple linear regression was used to examine whether artificial sweetener consumption frequency remained associated with profile membership after adjustment for the same demographic covariates. Variance inflation factors were examined to rule out problematic multicollinearity among the overlapping age, marital status, and employment covariates. Satistical significance was set at *p* < 0.05.

## 3 Results

Models specifying two to six profiles were estimated and compared across the four covariance structures described in the Methods. Fit statistics for the five best-fitting models are summarized in Table 1. The three-profile solution with varying variances and zero covariances demonstrated the lowest BIC (5,223.27) and acceptable classification quality (entropy = 0.72; minimum posterior probability = 0.76; BLRT *p* = 0.01), and was further supported by the theoretical interpretability of the identified profiles. Each profile contained a sufficient number of respondents (*n* = 76, 129, and 181, respectively), with each exceeding the recommended minimum of 5% of the total sample (19.7%, 33.4%, and 46.9%, respectively).

**Table 1:** Model fit statistics of latent profile analyses.

Latent profile model	BIC	AIC	Entropy	prob_min	BLRT p
Prof 3 Var varying Cov zero	5223.27	5096.68	0.72	0.76	0.01
Prof 2 Var varying Cov varying	5238.25	5076.06	0.58	0.86	0.01
Prof 4 Var equal Cov zero	5263.65	5152.89	0.76	0.85	0.01
Prof 3 Var varying Cov varying	5273.43	5028.17	0.65	0.70	0.01
Prof 5 Var equal Cov zero	5280.61	5146.11	0.73	0.50	0.03

### 3.1 Latent profile analysis results

Latent Profile Analysis (LPA) identified three distinct attitudes toward artificial sweeteners among participants. The model fit statistics indicated that the three-profile model with variable variance and zero covariance (Prof 3 Var varying Cov zero) was the most appropriate, as evidenced by the lowest Akaike Information Criterion (AIC = 5096.68) and a favorable Bayesian Information Criterion (BIC = 5223.27), along with an entropy value of 0.72, suggesting acceptable classification certainty. Each profile reflects unique characteristics based on the five dimensions of the Artificial Sweetener Attitude Scale (AASS). Profile 1 exhibited high acceptance and low perceived risk toward artificial sweeteners, while Profile 2 demonstrated some concerns about acceptance and about health risks. Profile 3 showed low acceptance and high perceived risk, indicating strong reservations regarding consumption.

### 3.2 Latent profile characteristics of artificial sweetener consumers

The latent profile analysis identified three distinct attitudinal profiles toward artificial sweeteners. Profile 1—Benefit-oriented consumers—showed the highest standardized scores on acceptance (Z ≈ 0.74) and perceived benefit (Z ≈ 1.21), the lowest scores on perceived risk (Z ≈ −0.91), and high trust in regulatory authorities (Z ≈ 0.74), with relatively low motivation for natural foods. Profile 2—Risk-sensitive consumers—exhibited the highest scores on perceived risk (Z ≈ 1.06) and motivation for natural foods (Z ≈ 0.64), alongside the lowest scores on acceptance (Z ≈ −0.65) and perceived benefit (Z ≈ −0.73), with near-zero trust. Profile 3—Neutral consumers—displayed near-zero standardized scores across all five subscales, reflecting attitudinal ambivalence rather than a clearly positive or negative orientation. As illustrated in Figure 2, Profiles 1 and 2 display near-mirror-image trajectories across the acceptance, risk, and benefit subscales, while Profile 3 consistently clusters near the zero line—a pattern further supported by the superiority of the three-profile solution over both simpler (two-profile: BIC = 5,238.25) and more complex (four-profile: BIC = 5,263.65) alternatives.

**Figure 2:**
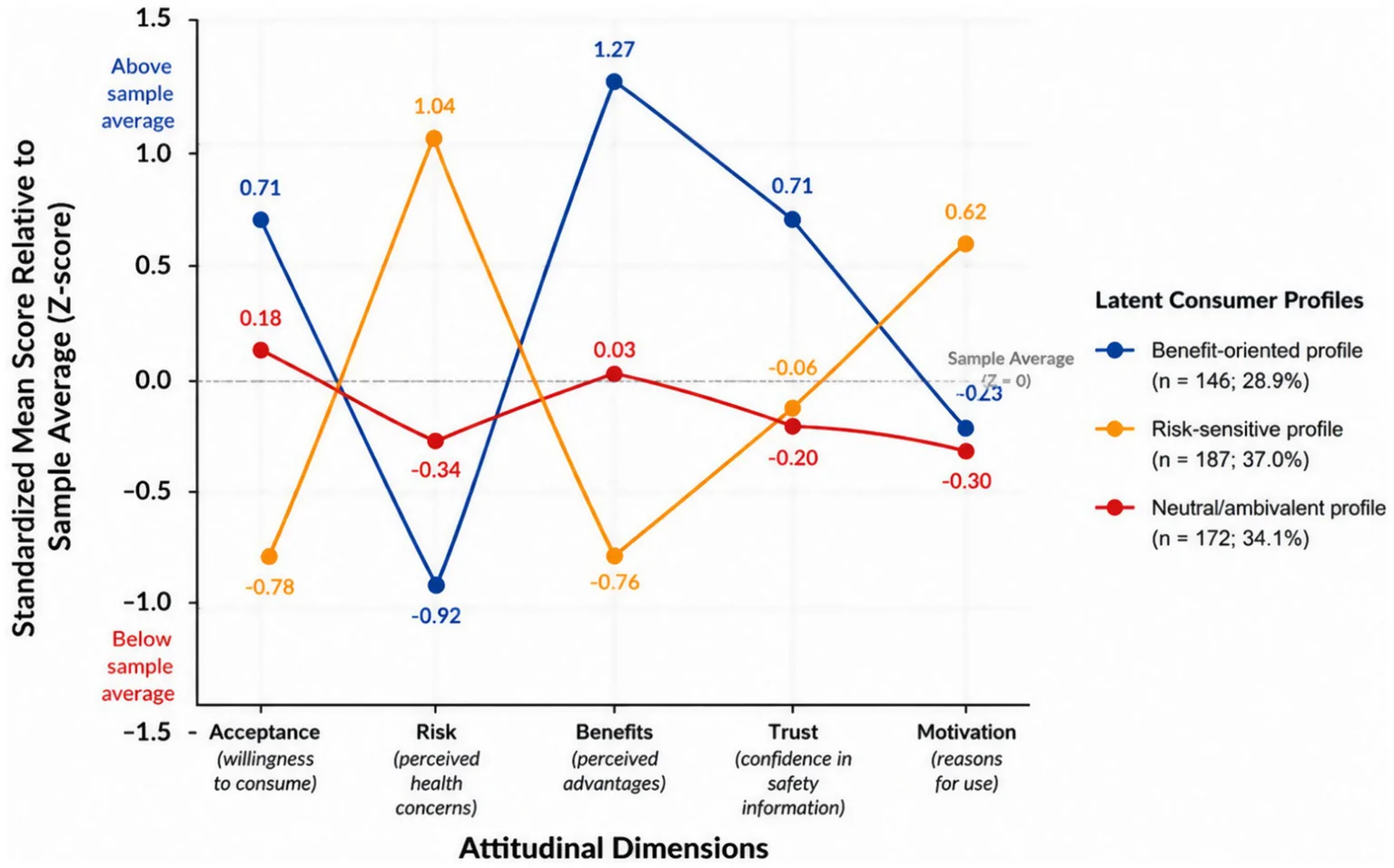
Latent attitudinal profiles toward artificial sweeteners across the three latent consumer profiles. Values above 0 indicate attitudes above the overall sample average, whereas values below 0 indicate attitudes below the sample average. The three latent profiles were identified using Latent Profile Analysis based on participants’ responses across five attitudinal dimensions: acceptance, perceived risk, perceived benefits, trust, and motivation toward artificial sweeteners.

#### 3.2.1 Socio-demographic characteristics across latent profiles

Table 2 presents the distribution of socio-demographic characteristics across the three latent profiles. Overall, no statistically significant differences were observed between profiles in terms of gender (*p* = 0.147), age group (*p* = 0.329), monthly household income (*p* = 0.142), region of residence (*p* = 0.162), marital status (*p* = 0.575), or employment status (*p* = 0.665). The absence of significant differences across these variables suggests that attitudinal profile membership is not primarily determined by conventional demographic characteristics. However, a statistically significant difference was found for educational level (*p* = 0.035). Participants with postgraduate qualifications (Master’s or Ph.D.) were most prevalent in Profile 3—Neutral consumers (40.33%), compared with Profile 2 (risk-sensitive) (37.98%) and Profile 1 (34.21%). Conversely, participants with high school education or less were most prevalent in Profile 1—Benefit-oriented consumers (22.37%) relative to Profiles 2 (17.05%) and 3 (15.47%). This pattern might indicate that higher educational attainment is associated with greater exposure to conflicting scientific information, which may foster attitudinal ambivalence rather than a clearly positive or negative evaluative stance toward artificial sweeteners.

**Table 2:** Socio-demographic characteristics between latent profiles.

Variables	Profile 1 benefit-oriented consumers (*n* = 76)	Profile 2 risk-sensitive consumers (*n* = 129)	Profile 3 neutral consumers (*n* = 181)	Test statistic	*p*-value
	*n* (%)	*n* (%)	*n* (%)		
Gender				χ^2^ = 3.84	0.147
Male	26 (34.21)	49 (37.98)	50 (27.62)		
Female	50 (65.79)	80 (62.02)	131 (72.38)		
Age (years)				χ^2^ = 6.91	0.329
18–29	52 (68.42)	74 (57.36)	98 (54.14)		
30–39	15 (19.74)	41 (31.78)	53 (29.28)		
40–49	8 (10.53)	11 (8.53)	24 (13.26)		
50–65	1 (1.32)	3 (2.33)	6 (3.31)		
Educational level				FFH	0.035
High school or less	17 (22.37)	22 (17.05)	28 (15.47)		
Diploma	3 (3.95)	12 (9.30)	9 (4.97)		
Bachelor’s degree	30 (39.47)	46 (35.66)	71 (39.23)		
Master’s or Ph D	26 (34.21)	49 (37.98)	73 (40.33)		
Monthly family income (SAR)				FFH	0.142
<5,000	45 (59.21)	71 (55.04)	105 (58.01)		
5,000– < 10,000	13 (17.11)	24 (18.60)	33 (18.23)		
10,000– < 20,000	10 (13.16)	24 (18.60)	36 (19.89)		
≥ 20,000	8 (10.53)	10 (7.75)	7 (3.87)		
Region				FFH	0.162
Central	8 (10.53)	14 (10.85)	14 (7.73)		
Western	55 (72.37)	87 (67.44)	111 (61.33)		
Eastern	4 (5.26)	7 (5.43)	8 (4.42)		
Northern	5 (6.58)	15 (11.63)	33 (18.23)		
Southern	4 (5.26)	6 (4.65)	15 (8.29)		
Marital status					
Single	52 (68.42)	80 (62.02)	112 (61.88)	χ^2^ = 1.10	0.575
Married	24 (31.58)	49 (37.98)	69 (38.12)		
Employment status				FFH	0.665
Student	39 (51.32)	60 (46.51)	88 (48.62)		
Public sector employee	18 (23.68)	35 (27.13)	46 (25.41)		
Private sector employee	7 (9.21)	14 (10.85)	14 (7.73)		
Self-employed	2 (2.63)	4 (3.10)	4 (2.21)		
Unemployed	10 (13.16)	16 (12.40)	29 (16.02%)		

Values are presented as frequency and percentage within each latent profile.

χ^2^ = Chi-square test; FFH = Fisher–Freeman–Halton exact test. *p* < 0.05 indicates statistical significance.

#### 3.2.2 Differences in artificial sweetener consumption across latent profiles

As shown in Table 3, significant differences in artificial sweetener consumption were observed across the three latent profiles (Kruskal–Wallis = 11.28, *p* = 0.004). The median consumption score was highest in Profile 1—Benefit-oriented consumers [29 (IQR: 17–41)], followed by Profile 3— Neutral consumers [25 (IQR: 14–39)], and lowest in Profile 2—Risk-sensitive consumers [22 (IQR: 12–33)]. This ordering is consistent with the attitudinal configurations of each profile: Profile 1’s elevated consumption aligns with its high perceived benefit and low perceived risk scores, which together reflect a favorable disposition toward artificial sweeteners that is likely to translate into greater actual use; conversely, Profile 2’s lower consumption is consistent with its high perceived risk and strong motivation for natural foods, representing attitudinal barriers to consumption. The analytical interpretation of these associations is developed further in the Discussion.

**Table 3:** Consumption differences between latent profiles.

Variables	Profile 1 benefit-oriented consumers (*n* = 76)	Profile 2 risk-sensitive consumers (*n* = 129)	Profile 3 neutral consumers (*n* = 181)	Test statistic	*P*-value
	*Median (interquartile range)*				
Consumption	29 (17, 41)	22 (12, 33)	25 (14, 39)	K–W = 11.28	0.004
	*n (%)*				
Consumption level				χ^2^ = 8.03	0.090
Low	21 (27.63)	54 (41.86)	57 (31.49)		
Moderate	23 (30.26)	42 (32.56)	63 (34.81)		
High	32 (42.11)	33 (25.58)	61 (33.70)		

Values for categorical variables are presented as frequency and percentage within the profile. K–W = Kruskal–Wallis test; χ^2^ = Chi-square test. *p* < 0.05 indicates statistical significance.

Regarding categorized consumption levels, a higher proportion of participants in Profile 1 were classified as high consumers (42.11%) compared to Profile 2 (25.58%) and Profile 3 (33.70%); however, these differences did not reach statistical significance (*p* = 0.090), likely reflecting loss of information through score categorization.

### 3.3 Factors associated with latent artificial sweetener consumption profiles and consumption frequency

Table 4 presents a fully adjusted multinomial logistic regression examining demographic factors associated with latent artificial sweetener consumption profile membership. The Neutral profile served as the reference category, and two contrasts were estimated simultaneously: Benefit-oriented versus Neutral and Risk-sensitive versus Neutral. After adjustment for sex, age, education, income, region, marital status, and employment, participants aged 30–39 years had lower odds of belonging to the Benefit-oriented profile than the Neutral profile (OR = 0.35, 95% CI: 0.14–0.86, *p* = 0.022). Participants from the Northern region also had lower odds of membership in both the Benefit-oriented (OR = 0.23, 95% CI: 0.08–0.67, *p* = 0.007) and Risk-sensitive (OR = 0.45, 95% CI: 0.21–0.96, *p* = 0.038) profiles compared with the Neutral profile. Participants aged 40–49 years had lower odds of Risk-sensitive versus Neutral membership (OR = 0.29, 95% CI: 0.10–0.86, *p* = 0.026). However, the overall multinomial model was not statistically significant (LR χ^2^(38) = 40.71, *p* = 0.352; pseudo-R^2^ = 0.051); therefore, the individual significant contrasts should be interpreted cautiously.

**Table 4:** Multinomial logistic regression predicting latent profile membership, fully adjusted for all demographic covariates (*n* = 386).

Predictor	Benefit-oriented profile (*n* = 76) vs. Neutral					Risk-sensitive profile (*n* = 129) vs. Neutral				
	B	SE	*p*	OR	95% CI	B	SE	*p*	OR	95% CI
Sex (ref. male)										
Female	−0.129	0.322	0.690	0.88	0.47–1.65	−0.366	0.270	0.175	0.69	0.41–1.18
Age (ref. 18-29 y.)										
30–39 y.	−1.057	0.462	0.022	0.35	0.14–0.86	−0.365	0.360	0.311	0.69	0.34–1.41
40–49 y.	−1.105	0.643	0.086	0.33	0.09–1.17	−1.225	0.550	0.026	0.29	0.10–0.86
50–65 y.	−2.230	1.265	0.078	0.11	0.01–1.28	−1.283	0.870	0.140	0.28	0.05–1.53
Education (ref. high school)										
Bachelor’s	−0.535	0.426	0.209	0.59	0.25–1.35	−0.270	0.378	0.474	0.76	0.36–1.60
Diploma	−0.438	0.763	0.566	0.65	0.14–2.88	0.775	0.553	0.161	2.17	0.73–6.42
Master’s/PhD	−0.643	0.460	0.162	0.53	0.21–1.29	−0.252	0.404	0.533	0.78	0.35–1.72
Income (SAR) (ref. <5,000)										
10,000–<20,000	−0.309	0.613	0.614	0.73	0.22–2.44	−0.008	0.484	0.987	0.99	0.38–2.56
5,000–<10,000	−0.039	0.464	0.933	0.96	0.39–2.39	−0.062	0.382	0.871	0.94	0.44–1.99
≥20,000	1.214	0.702	0.084	3.37	0.85–13.33	0.703	0.636	0.270	2.02	0.58–7.03
Region (ref. western)										
Central	0.150	0.506	0.766	1.16	0.43–3.13	0.207	0.430	0.630	1.23	0.53–2.86
Eastern	0.133	0.676	0.844	1.14	0.30–4.30	0.187	0.568	0.742	1.21	0.40–3.67
Northern	−1.469	0.543	0.007	0.23	0.08–0.67	−0.798	0.385	0.038	0.45	0.21–0.96
Southern	−0.632	0.623	0.310	0.53	0.16–1.80	−0.785	0.533	0.141	0.46	0.16–1.30
Marital status (ref. unmarried)										
Married	0.037	0.373	0.921	1.04	0.50–2.15	0.171	0.306	0.577	1.19	0.65–2.16
Employment (ref. student)										
Private sector	0.634	0.596	0.288	1.89	0.59–6.07	0.599	0.488	0.219	1.82	0.70–4.74
Public sector	0.919	0.609	0.131	2.51	0.76–8.27	0.661	0.496	0.183	1.94	0.73–5.12
Self-employed	0.836	0.969	0.389	2.31	0.35–15.41	0.714	0.776	0.358	2.04	0.45–9.36
Unemployed	0.213	0.473	0.653	1.24	0.49–3.13	0.046	0.396	0.907	1.05	0.48–2.28
Intercept	−0.042	0.391	0.914	—	—	0.127	0.356	0.721	—	—

Model fit: Pseudo R^2^ = 0.051; Log-likelihood = −381.62 (null = −401.97); LR χ^2^(38) = 40.71, *p* = 0.352; *N* = 386.

Table 5 presents a fully adjusted multiple linear regression examining whether latent profile membership was associated with the artificial sweetener consumption-frequency score, while controlling for demographic characteristics. Compared with the Neutral profile, membership in the Risk-sensitive profile was associated with a significantly lower consumption-frequency score (B = −6.510, SE = 2.047, t = −3.180, *p* = 0.002, 95% CI: −10.535 to −2.484). In contrast, the Benefit-oriented profile had a higher estimated consumption-frequency score than the Neutral profile, but this difference was not statistically significant (B = 3.738, *p* = 0.127). The overall regression model was statistically significant (*F*(22,363) = 2.535, *p* < 0.001), explaining 13.3% of the variance in consumption-frequency score (R^2^ = 0.133; adjusted R^2^ = 0.081).

**Table 5:** Multiple linear regression of consumption frequency score on latent profile membership, fully adjusted for all demographic covariates (*n* = 386).

Predictor	B	SE	*p*	OR	95% CI
Profile: Benefit-oriented (vs Neutral)	3.738	2.441	1.532	0.127	−1.062–8.538
Profile: Risk-sensitive (vs Neutral)	−6.510	2.047	−3.180	0.002	−10.535– −2.484
Sex (ref. male)					
Female	−3.125	2.013	−1.553	0.121	−7.082–0.833
Age (ref. 18-29 y.)					
30–39 y.	−3.565	2.699	−1.321	0.187	−8.872–1.742
40–49 y.	−5.424	4.104	−1.322	0.187	−13.496–2.647
50–65 y.	−5.633	6.571	−0.857	0.392	−18.556–7.290
Education (ref. high school)					
Bachelor’s	−1.280	2.750	−0.465	0.642	−6.689–4.129
Diploma	12.830	4.207	3.050	0.002	4.557–21.103
Master’s/PhD	−1.144	2.953	−0.387	0.699	−6.950–4.663
Income (SAR) (ref. <5,000)					
10,000–<20,000	−0.499	3.630	−0.137	0.891	−7.637–6.640
5,000–<10,000	2.637	2.851	0.925	0.355	−2.968–8.243
≥20,000	−4.617	4.469	−1.033	0.302	−13.405–4.171
Region (ref. western)					
Central	−3.723	3.210	−1.160	0.247	−10.035–2.589
Eastern	−0.857	4.266	−0.201	0.841	−9.247–7.532
Northern	2.885	2.862	1.008	0.314	−2.743–8.512
Southern	−4.999	3.776	−1.324	0.186	−12.426–2.427
Marital status (ref. unmarried)					
Married	0.345	2.288	0.151	0.880	−4.155–4.846
Employment (ref. student)					
Private sector	0.664	3.650	0.182	0.856	−6.513–7.841
Public sector	0.106	3.706	0.029	0.977	−7.181–7.393
Self-employed	−5.861	5.835	−1.004	0.316	−17.336–5.614
Unemployed	2.578	2.913	0.885	0.377	−3.151–8.307
Intercept	25.477	2.875	8.862	<0.001	19.823–31.130

Model fit: R^2^ = 0.133, Adjusted R^2^ = 0.081, F(22, 363) = 2.535, *p* < 0.001, *N* = 386.

## 4 Discussion

Understanding consumer attitudes toward artificial sweeteners is critical given the rising prevalence of obesity and metabolic disorders in the Gulf region and the increasing use of non-nutritive sweeteners as sugar substitutes (20–22). To date, the vast majority of studies conducted in this region have relied on variable-centered approaches that examine average associations across the population but cannot reveal the distinct configurations of beliefs, risk perceptions, and trust that coexist within different consumer subgroups. To our knowledge, no prior study has applied a person-centered analytical framework to characterize attitudinal profiles toward artificial sweeteners in a Gulf region and in Saudi Arabia. The present study addresses this gap by applying latent profile analysis to identify distinct attitudinal subgroups among Saudi adults—an approach that is novel in this regional context. The discussion that follows draws on the identified profile structure to propose specific, evidence-based directions for the design of targeted nutrition communication strategies and public health interventions in the Saudi context.

### 4.1 Identification and interpretation of latent profiles

This study identified three latent attitudinal profiles toward artificial sweeteners among Saudi adults: benefit-oriented consumers (19.7%), risk-concerned consumers (33.4%), and neutral consumers (46.9%). These profiles differed significantly across perceived benefits, perceived risks, trust in regulatory authorities, and motivation toward natural foods. Importantly, profiles were also significantly associated with continuous artificial sweetener consumption scores (*p* = 0.004), indicating that attitudinal differences corresponded with measurable behavioral variation. The novelty of this study lies in the use of a person-centered latent profile approach to examine artificial sweetener attitudes in a Middle Eastern population. Previous regional studies have primarily relied on variable-centered analyses describing isolated predictors of attitudes or consumption (21, 22). In contrast, the present study examined how multiple attitudinal dimensions co-occur within individuals, allowing identification of distinct consumer subgroups with differing behavioral patterns.

The benefit-oriented profile was characterized by high perceived benefits, lower perceived risks, stronger trust in regulatory authorities, and the highest median consumption score (29 [IQR: 17–41]). These findings suggest that positive evaluations of artificial sweeteners may be associated with more frequent use. Although motivations such as weight control or sugar reduction were not directly assessed in this study, previous Saudi and international studies have reported these factors as common reasons for consuming artificial sweeteners (8, 9). In contrast, the risk-sensitive profile showed the highest perceived risks, the strongest preference for natural foods, and the lowest median consumption score (22 [IQR: 12–33]). This pattern is consistent with literature showing that consumers with stronger concerns regarding food additives or artificial ingredients are less likely to consume products containing artificial sweeteners (40). The elevated preference for natural foods observed in this profile may reflect broader concerns about food processing and the artificiality of ingredients rather than artificial sweeteners specifically. The neutral profile demonstrated intermediate scores across attitudinal dimensions and consumption behavior. Given that this group represented nearly half of the sample (46.9%), the findings may indicate that many consumers hold neither strongly favorable nor strongly unfavorable attitudes toward artificial sweeteners. Such ambivalence may reflect uncertainty or exposure to mixed information regarding benefits and potential risks.

The identified profiles correspond with patterns reported in previous Saudi studies. For example, Alharthi et al. reported that some Saudi consumers perceived artificial sweeteners as beneficial for calorie reduction and weight management, whereas others expressed concerns regarding safety and preferred natural alternatives (21). These observations align, respectively, with the benefit-oriented and risk-concerned profiles identified in the present study. Similarly, Aljehany et al. (22) reported coexistence of positive perceptions regarding health benefits alongside concerns about long-term safety among Saudi adults. The current findings extend these observations by demonstrating that these attitudinal differences are associated with significantly different consumption levels. Specifically, benefit-oriented consumers reported higher continuous consumption scores than risk-sensitive consumers, while neutral consumers showed intermediate values. This behavioral gradient strengthens the interpretation that attitudinal heterogeneity is linked to actual consumption practices rather than representing only abstract perceptions. Therefore, the present findings contribute preliminary evidence that heterogeneous consumer profiles may also be relevant within Saudi dietary and cultural contexts.

### 4.2 Association between profiles and consumption behavior

A central finding of this study was the significant association between attitudinal profiles and continuous artificial sweetener consumption. This association represents an important contribution because previous regional studies have largely described perceptions or prevalence of use without examining whether distinct attitudinal configurations correspond to measurable differences in intake behavior. Benefit-oriented consumers reported the highest consumption levels, whereas risk-concerned consumers reported the lowest. This pattern is consistent with behavioral theories suggesting that individuals are more likely to engage in behaviors perceived as beneficial and less likely to engage in behaviors perceived as risky (41, 42). However, categorized consumption levels were not significantly associated with profile membership. This discrepancy may reflect reduced sensitivity caused by categorization of continuous data, which can obscure smaller behavioral differences between groups. The findings therefore suggest that attitudinal profiles may influence consumption along a gradient rather than through sharply distinct consumption categories. The observed differences across benefit-oriented, risk-sensitive, and neutral consumers also highlight the potential value of profile-tailored communication strategies. Specifically, the findings suggest that one-size-fits-all public health messaging may be insufficient, while targeted approaches addressing differing perceptions and concerns could support more effective behavioral interventions.

Importantly, the association between the risk-sensitive profile and lower consumption was robust across unadjusted, partially adjusted, and fully demographically adjusted specifications (Table 5), with the estimate remaining virtually unchanged (B = −5.77 unadjusted vs. B = −6.51 fully adjusted, p = 0.002) despite the inclusion of age, sex, income, region, marital status, and employment status. This consistency across specifications suggests that the lower consumption observed among risk-sensitive individuals is unlikely to be fully explained by demographic confounding and may instead reflect a genuine behavioral correspondence between risk-oriented attitudes and avoidance of artificial sweeteners. By contrast, the elevated consumption observed among benefit-oriented consumers did not reach statistical significance once demographic covariates were included, indicating that this association should be interpreted more cautiously and may be partly attributable to the demographic composition of this subgroup rather than attitudinal orientation alone.

### 4.3 Role of socio-demographic factors

The finding that most socio-demographic variables—including gender, age, marital status, employment status, monthly family income, and region of residence—were not significantly associated with latent profile membership warrants careful interpretation rather than being dismissed as a null result. One plausible explanation is that attitudes toward artificial sweeteners are shaped by psychological constructs—such as perceived risk, perceived benefit, and trust in regulatory institutions—that do not distribute neatly along demographic lines. This is consistent with a broader literature on food-related attitudes demonstrating that cognitive and attitudinal variables often outperform demographic predictors in explaining consumer heterogeneity (43, 44). It was specifically shown that beliefs, knowledge, and perceived control over health were more influential than sociodemographic characteristics in determining acceptance of functional foods—a finding that has been replicated across multiple food additive contexts, including artificial sweeteners (40, 43).

The sole exception to the general non-significance of demographic variables was educational level (*p* = 0.035), with individuals holding postgraduate qualifications more prevalent in the neutral profile. The sole exception to the general non-significance of demographic variables was educational level (*p* = 0.035), with individuals holding postgraduate qualifications more prevalent in the neutral profile. Although this finding suggests a potential association between educational attainment and attitudinal profile membership, its interpretation should be approached with caution. Previous research has shown that consumers may maintain elevated risk perceptions of sweeteners despite generally high levels of trust in regulatory authorities and institutional endorsements (45). The greater representation of highly educated individuals within the neutral profile may therefore reflect the complexity of consumer attitudes toward artificial sweeteners rather than a straightforward relationship between education and attitude direction. However, because the factors underlying profile membership were not directly assessed, the mechanisms explaining this association remain unclear and warrant further investigation. These interpretations, however, require direct empirical testing—for instance, through mediation analyses examining whether information diversity or scientific awareness mediates the relationship between education and profile membership. Taken together, the socio-demographic findings suggest that neither demographic segmentation alone nor education as a proxy for health literacy can adequately explain attitudinal differentiation toward artificial sweeteners in this population. Future research should incorporate psychological mediators to more rigorously identify the mechanisms underlying the profile structure identified here.

The multivariable analysis reported clarified this issue directly. When education was modeled simultaneously with age, sex, income, region, marital status, and employment status, its association with profile membership was no longer statistically significant in any contrast (all *p* ≥ 0.16), indicating that the bivariate relationship between education and profile membership was not independent of other demographic characteristics. This finding is consistent with the interpretation offered above—that education alone is an insufficient proxy for the psychological constructs (perceived risk, perceived benefit, trust) that more plausibly drive attitudinal segmentation. Instead, age (30–39 years) and region of residence (Northern region) emerged as independent, though modest, predictors of profile membership, with Northern-region participants disproportionately represented in the neutral profile. Given the small size of this regional subgroup (*n* = 53) and the fact that the overall multivariable model did not significantly outperform the null model (likelihood ratio χ^2^ *p* = 0.352), these associations should be regarded as exploratory signals warranting replication rather than established determinants of attitudinal profile membership.

### 4.4 Public health implications

The identification of distinct attitudinal profiles may help inform more targeted nutrition communication strategies. Because benefit-oriented, risk-sensitive, and neutral consumers differed in both perceptions and consumption levels, communication approaches addressing artificial sweeteners may need to be adapted according to consumer concerns and informational needs. This approach is consistent with evidence suggesting that tailored health communication strategies are generally more effective than uniform population-wide messaging because they account for differences in beliefs, motivations, and risk perceptions (46). For example, benefit-oriented consumers, who demonstrated the highest consumption levels, may benefit from guidance emphasizing moderation and evidence-based information regarding appropriate use. Consumers with favorable attitudes toward artificial sweeteners may underestimate potential uncertainties or rely heavily on perceived health benefits such as calorie reduction or weight control (47). Therefore, balanced communication strategies emphasizing both potential benefits and current evidence limitations may support more informed decision-making. In contrast, risk-sensitive consumers may respond more effectively to transparent communication addressing safety evaluation and regulatory oversight processes. Previous research has shown that trust in regulatory authorities is an important determinant of food technology acceptance and that transparent risk communication may reduce perceived uncertainty surrounding food additives (44, 48). In addition, acknowledging consumer concerns rather than dismissing them may improve communication credibility, particularly among individuals with stronger preferences for natural foods or skepticism toward processed ingredients (40). Neutral consumers may represent an important target for educational interventions because they displayed intermediate attitudes and consumption levels and constituted the largest subgroup in the sample. Individuals with less strongly established attitudes may be more receptive to educational messaging and behavioral interventions (49). Practical strategies could include simplified nutrition education materials, clearer front-of-package labeling, or public awareness campaigns explaining the role, regulation, and current evidence regarding artificial sweeteners. Front-of-package labeling and simplified nutrition information have previously been associated with improved consumer understanding and healthier food selection behaviors (50).

More broadly, these findings support the potential value of audience segmentation approaches in nutrition policy and health promotion. Consumer segmentation has been increasingly recommended in public health nutrition because it allows interventions to better align with the informational needs and behavioral characteristics of specific population subgroups (51). Within the Saudi context, where obesity and diet-related chronic diseases remain major public health concerns, integrating profile-based communication strategies into nutrition education campaigns may improve engagement and intervention relevance.

### 4.5 Limitations

Several methodological limitations of the present study warrant consideration when interpreting its findings. First, the cross-sectional design precludes causal inferences between attitudinal profiles and artificial sweetener consumption behaviors; the observed associations may reflect attitudes influencing consumption, consumption shaping attitudes, or reciprocal effects (52). Longitudinal or experimental designs are needed to establish temporal order. Second, the use of convenience sampling—while pragmatic—limits the generalizability of findings to the broader Saudi adult population. The sample overrepresented younger and more educated individuals, and probability-based sampling would be required to produce representative estimates. Consistent with this recruitment strategy, which was anchored at King Abdulaziz University in Jeddah, the sample was also geographically concentrated in the Western region of Saudi Arabia (67.4–72.4% of participants across profiles), and while region of residence did not differ significantly across latent profiles (*p* = 0.162), the identified profiles may not adequately represent attitudinal patterns among adults residing in other regions of the country (53). Furthermore, participants were recruited through social media platforms and university networks, which introduces self-selection bias and further restricts the representativeness of the sample to digitally connected and academically affiliated populations; this constraint should be acknowledged as an additional boundary condition on the generalizability of the findings (53). Third, self-reported data are subject to recall bias (inaccurate memory of consumption) and social desirability bias (underreporting of unfavorable attitudes or behaviors), both of which may distort the estimated associations (54, 55). Fourth, although Latent Profile Analysis (LPA) is a rigorous method, the resulting profiles depemodel-specification decisions (e.g., the, e.g., number of profiles, fit indices) and sample characteristics. The modest sample size (*N* = 386) and the smallest profile (*n* = 76) contribute to classification uncertainty (entropy = 0.72), reducing precision and statistical power. Replication in larger, independent samples is necessary to confirm the stability of the three-profile solution (25, 37). The overall scale reliability was borderline acceptable (Cronbach’s α = 0.68), and measurement imprecision may have attenuated the separability of latent profiles and introduced random error (56). An additional limitation relates to the Arabic version of the Artificial Sweetener Attitude Scale (AASS). Although the instrument underwent translation, back-translation, expert review, and reliability assessment, a comprehensive psychometric validation—including exploratory or confirmatory factor analysis and measurement invariance testing—was not performed. Consequently, the construct validity of the Arabic version cannot be considered fully established, and future research should undertake formal validation of the instrument in Arabic-speaking populations. Finally, the absence of direct measures of motivations (e.g., diagnosed diabetes, prediabetes, obesity, weight-loss efforts, taste preference) and contextual factors (e.g., food environment, marketing exposure) limits the ability to explain the mechanisms underlying the observed profile differences; interpretations offered are therefore hypothetical and require empirical testing.

## 5 Conclusion

This study identified three distinct attitudinal profiles toward artificial sweeteners among Saudi adults and demonstrated that these profiles were significantly associated with differences in continuous consumption behavior. Benefit-oriented consumers reported higher consumption levels, whereas risk-sensitive consumers reported lower levels, with neutral consumers occupying an intermediate position. Multivariable analysis further strengthened confidence in this pattern, as the reduced consumption observed among risk-sensitive consumers remained statistically significant after adjustment for age, sex, income, region, marital status, employment status, indicating that this attitude–behavior association is unlikely to be explained by demographic confounding alone. In contrast, educational attainment—the only socio-demographic factor associated with profile membership in bivariate analysis—did not remain an independent predictor once other demographic factors were accounted for, underscoring that psychological rather than purely demographic factors likely underlie attitudinal segmentation in this population. The findings contribute to the literature by applying a person-centered analytical approach within a Saudi population, highlighting the heterogeneity of consumer perceptions toward artificial sweeteners. Rather than viewing consumers as a homogeneous group, the results suggest that attitudes toward artificial sweeteners vary with perceived benefits and risks, trust in regulatory authorities, and preference for natural foods. From a public health perspective, these findings support the potential value of more targeted nutrition communication strategies. In particular, the large proportion of neutral consumers suggests that a substantial segment of the population may be receptive to clearer and evidence-based educational messaging regarding artificial sweeteners and their role within healthy dietary patterns.

## Data Availability

The raw data supporting the conclusions of this article will be made available by the authors, without undue reservation.
