# Supplementary material for: A latent profile analysis of attitudes towards artificial sweetener consumption among adults in Saudi Arabia
Source: Front Public Health. 2026 Oct 9;14:1841996. doi: 10.3389/fpubh.2026.1841996 (PMC13690729; doi:10.3389/fpubh.2026.1841996)
Supplement: Supplementary file 1 [file Data_Sheet_1.PDF]

Appendix (1) Survey forms

Consumption Patterns and Attitudes towards Artificial Sweeteners among Saudi Adults: A cross-sectional study

أنماط الاستهلاك والمواقف تجاه المحليات الصناعية لدى البالغين السعوديين: دراسة مقطعية

By

FADHAH MOHAMMED ALATWI

A thesis submitted for the requirements of the degree of Master
(Food and Nutrition/Nutrition Sciences)

Supervised by

Dr. Buthaina Aljehany

Dr. Eman Abduljawad

1446 Hejri / 2024-2025 AC

أنت مدعو للمشاركة في استبيان ضمن دراسة بحثية لمتطلبات درجة الماجستير في التغذية وعلم الأغذية بجامعة الملك عبد العزيز. تهدف الدراسة لتقييم أنماط استهلاك ومواقف المحليات الصناعية. الاستبيان يشمل أقسامًا حول البيانات الديموغرافية والمواقف تجاه المحليات وأنماط الاستهلاك.

You are invited to participate in a survey as part of a research study required for a Master's degree in Food and Nutrition at King Abdulaziz University. The study aims to assess consumption patterns and attitudes towards artificial sweeteners. The questionnaire includes sections on demographic data, attitudes toward sweeteners, and consumption patterns.

هل أنت موافق/ة على المشاركة في الاستبيان: ☐ نعم☐ لا

Do you agree to participate in the survey: ☐ Yes☐ No

الأقسام الرئيسية للاستبيان

Main Sections of the Questionnaire

البيانات الديموغرافية والاجتماعية

Demographic and Social Data

الجنس: ☐ ذكر ☐ أنثى<br>Gender: ☐ Male ☐ Female
العمر: ☐ 18-29 ☐ 30-39 ☐ 40-49 ☐ 50-65<br>Age: ☐ 18-29 ☐ 30-39 ☐ 40-49 ☐ 50-65
المستوى التعليمي: ☐ الثانوية أو أقل ☐ الدبلوم ☐ البكالوريوس ☐ دراسات عليا<br>Educational Level: ☐ High School or lower ☐ Diploma ☐ Bachelor's degree ☐ Postgraduate
الحالة الاجتماعية: ☐ غير متزوج ☐ متزوج<br>Marital Status: ☐ Unmarried ☐ Married
الحالة الوظيفية: ☐ طالب ☐ موظف في القطاع العام ☐ موظف في القطاع الخاص ☐ أعمال حرة ☐ بدون وظيفة<br>Employment Status: ☐ Student ☐ Public sector employee ☐ Private sector employee ☐ Self-employed ☐ Unemployed
الدخل الشهري: ☐ أقل من 5000 ريال ☐ بين 5000 و10000 ريال ☐ بين 11000 و20000 ريال ☐ أكثر من 20000 ريال<br>Monthly Income: ☐ Less than 5000 SAR ☐ Between 5000 and 10000 SAR ☐ Between 11000 and 20000 SAR ☐ More than 20000 SAR
المنطقة: ☐ الوسطى ☐ الغربية ☐ الشرقية ☐ الشمالية ☐ الجنوبية<br>Region: ☐ Central ☐ Western ☐ Eastern ☐ Northern ☐ Southern

أسباب استهلاك المحليات الصناعية خلال الشهر الماضي: ☐ داء السكري و/أو حالة طبية ☐اتباع نظام غذائي لفقدان الوزن ☐جزء من أسلوب حياة صحي ☐أخرى<br>Reasons for consuming artificial sweeteners in the past month: ☐ Diabetes and/or medical condition☐ Weight loss diet☐ Part of a healthy lifestyle☐ Other

كم مرة تقرأ الملصقات الغذائية للتحقق من وجود المحليات الصناعية: ☐ دائمًا☐ غالبًا☐ أحيانًا☐ نادرًا☐ أبدًا<br>How often do you read food labels to check for the presence of artificial sweeteners: ☐ Always☐ Often☐ Sometimes☐ Rarely☐ Never
هل تعاني من الأمراض التالية؟ (بيلة الفينيل كيتون أو اضطرابات الأكل): ☐ نعم☐ لا<br>Do you suffer from the following diseases? (Phenylketonuria or eating disorders): ☐ Yes☐ No

Attitudes Towards Artificial Sweeteners
المو اقف اتجاه المحليات الصناعية

Agree very strongly=1	Agree Strongly=2	Agree=3	Disagree=4	Disagree Strongly=5	Disagree very strongly=6
موافق بشدة عالية=1	موافق بشدة=2	موافق=3	غير موافق=4	غير موافق بشدة=5	غير موافق بشدة عالية=6

Item<br>البند						
**Acceptance**<br>**التقبل**						
I think it is unimportant to check on the packaging whether a food contains artificial sweeteners.<br>أعتقد أنه من غير المهم التحقق على العبوة مما إذا كان الطعام يحتوي على محليات صناعية	1	2	3	4	5	6
I have more important things to do than worry about artificial sweeteners.<br>لدي أشياء أكثر أهمية للقيام بها من الاهتمام بشأن المحليات الصناعية	1	2	3	4	5	6
I can accept that certain foods contain artificial sweeteners.<br>يمكنني تقبل أن بعض الأطعمة تحتوي على محليات صناعية	1	2	3	4	5	6
People give too much thought to artificial sweeteners.<br>يفكر الناس كثيرا في المحليات الصناعية	1	2	3	4	5	6
Artificial sweeteners cannot be harmful; otherwise, they would not be contained in so many foods.<br>المحليات الصناعية لا يمكن أن تكون ضارة. وإلا لن يتم احتواؤها في العديد من الأطعمة	1	2	3	4	5	6
It does not bother me if my foods contain artificial sweeteners.<br>لا يزعجني إذا كانت أطعمتي تحتوي على محليات صناعية	1	2	3	4	5	6
**Risk**<br>**المخاطر المدركة**						
When I think of artificial sweeteners, I get an uneasy feeling.	1	2	3	4	5	6

Item<br>البند						
أشعر بعدم الارتياح عندما أفكر في المحليات الصناعية						
I am worried about what effects artificial sweeteners could have on my body.<br>أنا قلق بشأن التأثيرات التي يمكن أن تسببها المحليات الصناعية على جسدي	1	2	3	4	5	6
I think that certain artificial sweeteners are unhealthy.<br>أعتقد أن بعض المحليات الصناعية غير صحية	1	2	3	4	5	6
I think that certain artificial sweeteners are harmful to health.<br>أعتقد أن بعض المحليات الصناعية ضارة بالصحة	1	2	3	4	5	6
I think that artificial sweeteners are a risk to human health.<br>أعتقد أن المحليات الصناعية تشكل خطرا على صحة الإنسان	1	2	3	4	5	6
Benefits<br>الفوائد						
Artificial sweeteners allow for a reduction of unnecessary calories.<br>استخدام المحليات الصناعية يساهم في خفض السعرات الحرارية المتناولة	1	2	3	4	5	6
Artificial sweeteners bring about many benefits for consumers.<br>تجلب المحليات الصناعية العديد من الفوائد للمستهلكين	1	2	3	4	5	6
If artificial sweeteners did not exist, many diet products could not be produced.<br>إذا لم تكن المحليات الصناعية موجودة، فلا يمكن إنتاج العديد من منتجات الحمية	1	2	3	4	5	6
The use of artificial sweeteners brings benefits for me personally.<br>إن استخدام المُحليات الصناعية يجلب لي فوائد شخصية	1	2	3	4	5	6
Artificial sweeteners allow for indulgence without regret.<br>تسمح المحليات الصناعية بالاستمتاع دون ندم	1	2	3	4	5	6
Trust in Regulators<br>الثقة في الهيئات التنظيمية						
I trust the regulators (such as the Food and Drug Authority) to make sure every necessary step is taken to protect consumers' health.<br>أثق في الهيئة التنظيمية (الهيئة السعودية للغذاء والدواء) من التأكد من اتخاذ كل الخطوات الضرورية لحماية صحة المستهلكين	1	2	3	4	5	6
I think that you can trust the regulators (such as the Food and Drug Authority).<br>أعتقد أنه يمكنك الوثوق في الهيئة التنظيمية (الهيئة السعودية للغذاء والدواء)	1	2	3	4	5	6

Item<br>البند						
I trust the regulators (such as the Food and Drug Authority) in relation to the licensing and control of artificial sweeteners in foods.<br>أثق في الهيئة التنظيمية (الهيئة السعودية للغذاء والدواء) فيما يتعلق بترخيص ومراقبة المحليات الصناعية في الأطعمة	1	2	3	4	5	6
Motivation for Natural Foods<br>الدافع للأطعمة الطبيعية						
Natural foods are better for my health.<br>الأطعمة الطبيعية أفضل لصحتي	1	2	3	4	5	6
I feel good when I eat natural foods.<br>أشعر أنني بحالة جيدة عندما أتناول الأطعمة الطبيعية	1	2	3	4	5	6
I gladly pay a higher price for natural foods.<br>أنا سعيد بدفع سعر أعلى للأطعمة الطبيعية	1	2	3	4	5	6
The more natural the products are, the higher the quality of nutrients and vitamins.<br>كلما كانت المنتجات طبيعية، زادت جودة العناصر الغذائية والفيتامينات	1	2	3	4	5	6
Natural foods taste better than other foods.<br>الأطعمة الطبيعية طعمها أفضل من الأطعمة الأخرى	1	2	3	4	5	6
I pay attention during grocery shopping to ensure that the foods are as natural as possible.<br>أنتبه أثناء التسوق للتأكد من أن الأطعمة طبيعية قدر الإمكان	1	2	3	4	5	6

Consumption Patterns: Frequency of Consumption Questionnaire

أنماط الاستهلاك: استبيان تكرار الاستهلاك

During the past month, how many times did you consume the following products that contain artificial sweeteners?<br>خلال الشهر الماضي، كم مرة استهلكت المنتجات التالية والمضاف إليها المحليات الصناعية؟	Never<br>أبدا	Once a month<br>1 مرة في الشهر	2-3 times a month<br>3-2 مرات في الشهر	1-2 times a week<br>2-1 مرات في الأسبوع	3-4 times a week<br>4-3 مرات في الأسبوع	5-6 times a week<br>6-5 مرات في الأسبوع	Once a day<br>1 مرة في اليوم	2-3 times a day<br>3-2 مرات في اليوم	4-5 times a day<br>5-4 مرات في اليوم	6 times or more a day<br>6 مرات في اليوم
Beverages<br>المشروبات										
Beverage with artificial sweeteners like Light Tang.<br>مشروب مضاف إليه محليات صناعية مثل لايت تانغ										
Chocolate beverages with artificial sweeteners like sugar-free Nestlé and Nesquik.<br>مشروبات الشوكولاتة مضاف إليها محليات صناعية مثل نستله الخالية من السكر، نسكويك										
Iced diet tea with artificial sweeteners like instant Lepton iced tea and ready-to-drink Lepton sugar-free tea.<br>شاي دايت المثلج مضاف إليه محليات صناعية مثل مزيج شاي مثلج سريع التحضير ليبتون، شاي ليبتون خالي من السكر الجاهز للشرب										
Carbonated drinks with artificial sweeteners like Coca-Cola Zero, Seven Up Diet, Pepsi Diet, Dew Diet, and Vimto Fizzy Diet.<br>مشروبات غازية مضاف إليها محليات صناعية مثل كوكاكولا زيرو، سفن اب دايت، ببسي دايت، ديو دايت، فيمتو فزي دايت										
Energy drinks with artificial sweeteners like Red Bull.										

During the past month, how many times did you consume the following products that contain artificial sweeteners? خلال الشهر الماضي، كم مرة استهلكت المنتجات التالية والمضاف إليها المحليات الصناعية؟	Never أبدا	Once a month 1 مرة في الشهر	2-3 times a month 3-2 مرات في الشهر	1-2 times a week 2-1 مرات في الأسبوع	3-4 times a week 4-3 مرات في الأسبوع	5-6 times a week 6-5 مرات في الأسبوع	Once a day 1 مرة في اليوم	2-3 times a day 3-2 مرات في اليوم	4-5 times a day 5-4 مرات في اليوم	6 times or more a day 6 مرات في اليوم
مشروبات الطاقة المضاف إليها محليات صناعية مثل ريد بول										
Ready-to-drink light coffee with artificial sweeteners like Light Frappuccino. قهوة لايت جاهزة للشرب مضاف إليها محليات صناعية مثل فرابوتشينو لايت										
Hot beverages with artificial sweeteners like tea and coffee. مشروب ساخن مضاف إليه محليات صناعية مثل الشاي والقهوة										
Cooked Food الطعام المطبوخ										
Cooked grains with artificial sweeteners like waffles and pancakes. حبوب مطبوخة مضاف إليها محليات صناعية مثل الوافل، البان كيك										
Ready-to-eat cold cereals with artificial sweeteners like Nestlé cornflakes. حبوب باردة جاهزة للأكل مضاف إليها محليات صناعية مثل رقائق الذرة نستله										
Spreadable Foods الأغذية القابلة للدهن										
Fruit jams with artificial sweeteners. مربى الفواكه مضاف إليها محليات صناعية										
Spreadable chocolate with artificial sweeteners.										

During the past month, how many times did you consume the following products that contain artificial sweeteners?<br>خلال الشهر الماضي، كم مرة استهلكت المنتجات التالية والمضاف إليها المحليات الصناعية؟	Never<br>أبدا	Once a month<br>1 مرة في الشهر	2-3 times a month<br>3-2 مرات في الشهر	1-2 times a week<br>2-1 مرات في الأسبوع	3-4 times a week<br>4-3 مرات في الأسبوع	5-6 times a week<br>6-5 مرات في الأسبوع	Once a day<br>1 مرة في اليوم	2-3 times a day<br>3-2 مرات في اليوم	4-5 times a day<br>5-4 مرات في اليوم	6 times or more a day<br>6 مرات في اليوم
شوكولاتة قابلة للدهن مضاف إليها محليات صناعية										
Dairy Products<br>منتجات الألبان										
Rice pudding or custard with artificial sweeteners.<br>بودنغ الأرز أو الكاسترد مضاف إليه مُحليات صناعية										
Light yogurt with artificial sweeteners like Activia blackberry.<br>الزبادي لايت مضاف إليه محليات صناعية مثل أكتيفيا توت العليق										
Light ice cream with artificial sweeteners like Baskin-Robbins.<br>آيس كريم لايت مضاف إليه مُحليات صناعية مثل باسكن روبنز										
Biscuits and Sweets<br>البسكويت والحلوى										
Biscuits with artificial sweeteners like Digestive biscuits.<br>بسكويت مضاف إليه محليات صناعية مثل بسكويت دايجستيف										
Chocolate with artificial sweeteners like Crispy Cadbury.<br>شوكولاتة مضاف إليها محليات صناعية مثل كاندرل كرسبي										
Peppermint tablets with artificial sweeteners like Mentos and Strepsils<br>أقراص النعناع مضاف إليها محليات صناعية مثل سمنت وستربسيلز .										
Chewing gum with artificial sweeteners like Extra or Nova.<br>علكة مضاف إليها محليات صناعية مثل علكة اكسترا أو نوفا										
